# Second Spontaneous Opening and Closing of a Macular Hole Associated With High Altitude Following Pars Plana Vitrectomy: A Case Report

**DOI:** 10.7759/cureus.90804

**Published:** 2025-08-23

**Authors:** Xinyi Zuo, Lin Xie

**Affiliations:** 1 Ophthalmology, The Second Affiliated Hospital, Jiangxi Medical College, Nanchang University, Nanchang, CHN

**Keywords:** full-thickness macular hole, high-altitude retinopathy, pars plana vitrectomy, retinal detachment, spontaneous closure

## Abstract

A 60-year-old male with macula-off rhegmatogenous retinal detachment (RRD) underwent pars plana vitrectomy (PPV) in 2020. After surgery, the patient lived for several months in a high-altitude region (average altitude: 2,500 m/8,202 ft). One year later, optical coherence tomography (OCT) revealed a spontaneously formed full-thickness macular hole (FTMH), along with perifoveal cystoid changes and an epiretinal membrane (ERM). The patient stayed in Nanchang (average altitude of about 24 m/78.74 ft) over the following year, and the macular hole (MH) closed spontaneously, accompanied by an improvement in visual acuity. However, in 2023, when the patient returned to the high-altitude region, the MH reappeared. A second spontaneous closure was observed one year later.

Multiple mechanisms may contribute to the formation and closure of MHs after vitrectomy. Given these mechanisms, and the spontaneous closure twice observed in this patient, a conservative approach may be warranted - especially when cystoid changes and minimal traction are present. This differs from the traditional approach of immediate surgical intervention and suggests that individualized patient factors should be considered when making treatment decisions.

## Introduction

Full-thickness macular holes (FTMHs) are defects of all retinal layers in the fovea that can lead to significant visual impairment. They are most commonly associated with aging, trauma, and retinal detachment [1]. FTMH following pars plana vitrectomy (PPV) is an uncommon complication, and spontaneous closure is even more rarely observed. Although FTMH formation after PPV has been previously reported, repeated spontaneous closures without surgical intervention, particularly in post-vitrectomy eyes, remain exceedingly rare [2-6].

High-altitude retinopathy (HAR) encompasses a spectrum of retinal changes that occur in response to hypoxic conditions at high altitudes. Changes in retinal hemodynamics and disruption of the blood-retinal barrier (BRB) may contribute to vasogenic edema, inflammation, and oxidative stress [7]. The most frequently reported finding is retinal hemorrhage; less commonly, retinal vein occlusion may occur [8]. Macular hole (MH) formation triggered by changes in altitude is unusual. To the best of our knowledge, only two cases have been reported in a high-altitude region, as documented in a survey conducted in Nepal [9]. Understanding the pathophysiological mechanisms behind altitude-associated MH formation and spontaneous closure may provide valuable insights into its natural history and inform future management strategies.

In this report, we present the clinical course of a patient with a history of prolonged stays in a high-altitude environment, who developed and experienced spontaneous resolution of FTMH on two separate occasions following PPV for rhegmatogenous retinal detachment (RRD). We explore possible contributing mechanisms and discuss the rationale for a conservative, observation-based management approach in selected cases.

## Case presentation

**Figure 1:**
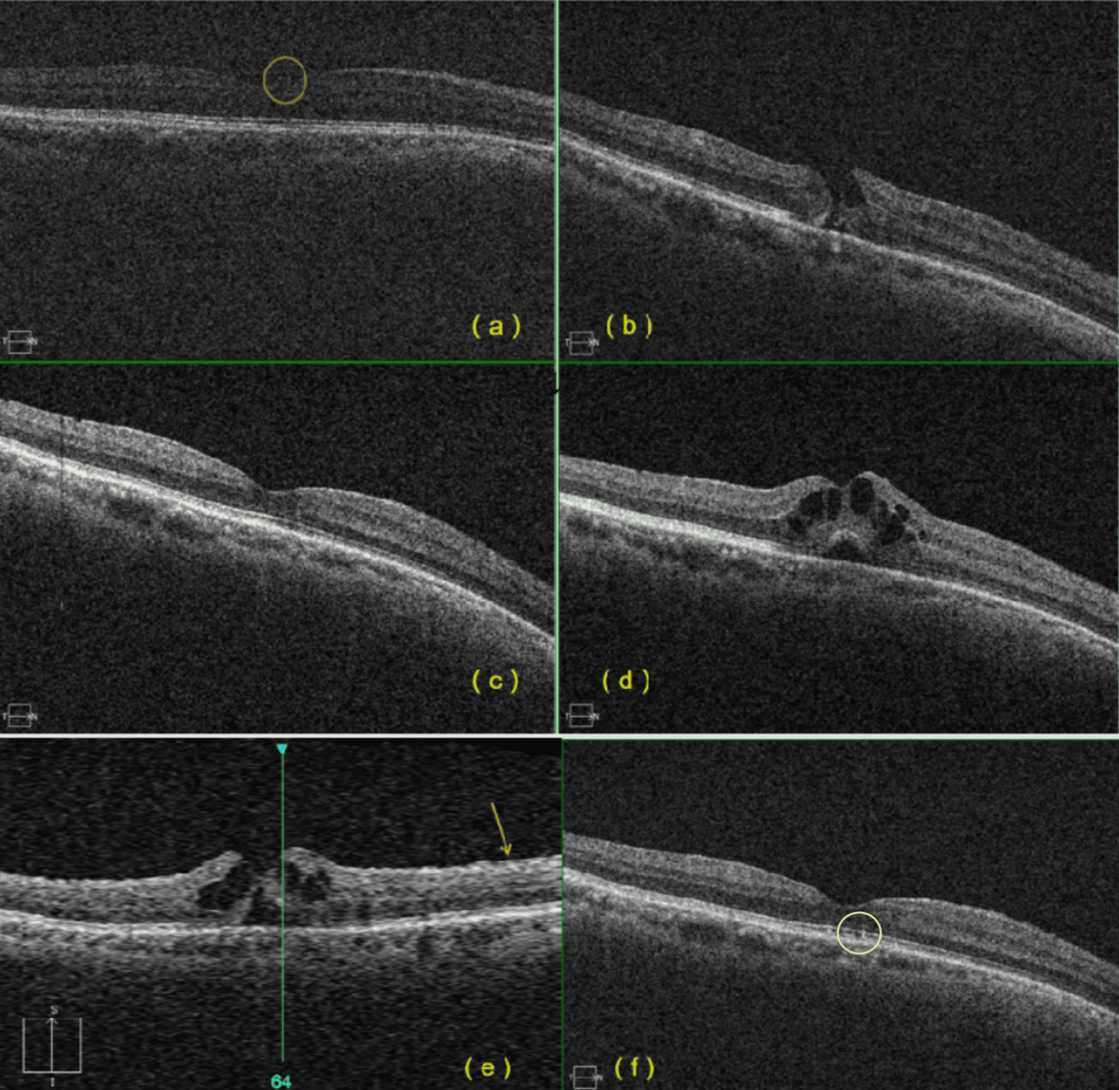
Optical coherence tomography (OCT) scans show the sequence of macular hole (MH) formation, closure, and reopening in the patient with macula-off retinal detachment post-vitrectomy. (a) OCT shows posterior hyaloid detachment and vitreomacular traction before silicone oil removal surgery (highlighted by the yellow circle). (b) In July 2021, OCT of the central fovea in the right eye reveals a full-thickness MH with cystic spaces in the parafoveal walls and defects in the ellipsoid zone (EZ), indicated by gaps in the EZ and interdigitation zone lines. (c) After 1 year of observation, OCT demonstrates normal foveal contour and spontaneous MH closure. (d, e) In September 2023, OCT images show recurrent large cystic cavities in the foveal walls, with subretinal fluid and detachment from the retinal pigment epithelium (yellow arrow notes the epiretinal membrane). (f) In May 2024, OCT after the second spontaneous MH closure shows a near-normal appearance of the inner layer, outer nuclear layer, and external limiting membrane, with a small defect in the central photoreceptor layer, marked by a gap in the EZ line (highlighted by the white circle). The image has been anonymized to protect the patient’s identity.

In January 2020, a 60-year-old man was diagnosed with macula-off RRD in his right eye. At the time of diagnosis, his best-corrected visual acuity (BCVA) was 20/50. The patient underwent PPV with retinal drainage, gas-fluid exchange, and silicone oil tamponade, followed by approximately one month of face-down positioning. In June 2020, six months later, the silicone oil was removed. Optical coherence tomography (OCT) imaging performed prior to silicone oil removal was of poor quality due to interference from the oil; however, some vitreous traction in the fovea could be identified (Figure 1a). One week after oil removal, the patient’s BCVA decreased to 20/160, despite the retina remaining attached. Soon after, the patient relocated to Yunnan Province, a high-altitude region with an average elevation of approximately 2,500 m (8,202 ft).

In July 2021, the patient returned with complaints of blurred vision and metamorphopsia. His BCVA had declined to 20/300. OCT revealed an FTMH, accompanied by cystic spaces in the parafoveal walls and slight elevation of the foveal edges (Figure 1b). Although macular surgery was initially considered, a conservative observational approach was adopted due to the patient’s reluctance to undergo another procedure. While residing in Nanchang - located at an average altitude of approximately 24 m (78.7 ft) - over the following year, the MH spontaneously closed, and BCVA improved to 20/200. OCT confirmed spontaneous closure of the FTMH and revealed epiretinal membrane (ERM) formation, likely resulting from glial cell proliferation (Figure 1c).

In the following year, the MH reopened but once again closed spontaneously within a year, coinciding with the patient’s relocation between Nanchang and Yunnan Province. By September 2023, the patient experienced a recurrence of symptoms, and his BCVA declined further. OCT revealed a reappearance of the FTMH, now accompanied by cystoid macular edema, subfoveal fluid, and sharply demarcated foveal edges (Figures 1d-1e). The previously noted ERM over the nerve fiber layer was contributing to retinal traction. Given the patient’s history of multiple spontaneous closures, a conservative observational approach was again selected. Follow-up OCT showed restoration of the normal foveal contour and improvement in visual function, although perifoveal cystoid changes persisted. By May 2024, the patient demonstrated significant visual improvement, and OCT confirmed spontaneous closure of the MH (Figure 1f).

## Discussion

A review of the literature reveals approximately 30 cases of spontaneous MH closure, 10 of which had a history of RRD. The remaining cases are mainly associated with high myopia or idiopathic MHs, and few reports describe recurrent MH formation (Table 1) [2-6]. Our patient’s case is unique in that he experienced two spontaneous closures following FTMH formation with cystoid changes, and both closures occurred after changes associated with high altitude (Figure 1). This case suggests that factors beyond hole size, such as the presence of an ERM or macular edema, may influence spontaneous opening and closure.

**Table 1:** Clinical characteristics and diagnosis of reported cases with spontaneous closure and recurrence of macular holes. BCVA, best-corrected visual acuity; OCT, optical coherence tomography; MH, macular hole; RRD, rhegmatogenous retina detachment; VH, vitreous hemorrhage; FTMH, full-thickness macular hole; PPV, pars plana vitrectomy; ILM, inner limiting membrane; CF, counting finger; C3F8, perfluoropropane

Case (first author, year)	Baseline BCVA	Primary disease	OCT/fundus photography demonstration	Spontaneous MH closure time, final BCVA	Management of recurrence MH
Kokame and McCauley (2002) [2]	20/70	MH	Stage 3 FTMH	2 years, 20/20	PPV + C3F8
Golan and Barak (2015) [3]	20/30	High myopia MH	Stage 4 FTMH	1 month, 20/25	Observation
Sridhar et al. (2017) [4]	20/30	Macular-off RRD	Stage 4 FTMH	1 month, 20/60	ILM peering
Komi et al. (2023) [5]	CF/30 cm	VH, RRD	Stage 2 FTMH	2 weeks, 20/28	PPV + ILM peering
Yagura et al. (2024) [6]	20/40	MH	Stage 4 FTMH	19 days, 20/50	C3F8

**Figure 2:**
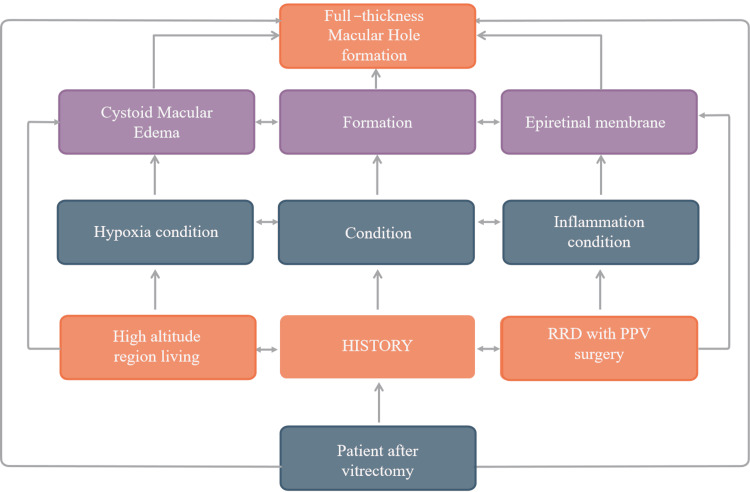
Potential mechanism of FTMH formation in patients after vitrectomy. Image credit: Xinyi Zuo FTMH, full-thickness macular hole; PPV, pars plana vitrectomy; RRD, rhegmatogenous retinal detachment

One potential cause is the promotion of ERM formation by cellular proliferation. In one study, glial cell proliferation is inhibited by the vitreous body and resumes after this inhibition is removed [10]. However, if the hole margins increase, glial migration may stop, causing tangential traction and further enlarging retinal defects [11]. Additionally, ERM may contribute to vitreofoveal traction, leading to the development of a subfoveal cyst and MH formation. Our patient also exhibited ERM formation with an FTMH (Figure 1e). The process is described in Figure 2.

Macular edema is another potential mechanism, with several cases of bilateral macular edema reported in high-altitude regions [12,13]. This reflects the fact that an increase in altitude leads to an increase in blood viscosity, which decreases oxygen transport capacity and triggers cellular swelling by stimulating the production and release of inflammatory factors that damage the inner BRB [14]. Furthermore, cystoid macular edema may place additional strain on the retinal pigment epithelium (RPE), which is primarily responsible for dehydration, a process that, in turn, promotes FTMH formation [15]. Figure 2 illustrates this process. However, more pathological evidence is needed to verify the occurrence of this mechanism.

Closure remains incompletely understood. After our patient returned to a low-altitude area, his hypoxia was relieved, and his cystoid edema resolved spontaneously as the functioning of his RPE cells recovered. In some cases, Müller cells play a crucial role in maintaining the structural integrity of the parafoveal region, possibly facilitating MH closure through their contractile properties and causing centripetal movement of the photoreceptor layer [16]. However, the mechanisms underlying their role require further investigation through pathology and imaging research.

## Conclusions

In conclusion, spontaneous MH formation and closure, particularly in patients with previously vitrectomized eyes and a history of travel to high-altitude regions, is a rare occurrence and can result in a favorable prognosis. Although surgical interventions are typically effective in treating MHs, they may not prevent recurrence. The case reported here emphasizes the importance of vigilant monitoring and imaging in patients with recurrent MHs post-vitrectomy. Additionally, individuals who have a history of ascending to high-altitude regions may develop HAR, leading to visual impairment. Therefore, a detailed ocular examination with a proper history is needed for all such patients. Given the spontaneous closure observed twice in this patient, a conservative approach may be warranted, especially when cystoid changes and minimal traction are present. This differs from the traditional approach of immediate surgical intervention and suggests that individualized patient factors should be considered when making treatment decisions.

A limitation of this case report is that it examined one patient, making it challenging to generalize the findings to a wider population. Additionally, although OCT imaging provided clear documentation of MH closure, more advanced imaging techniques might have yielded deeper insights into the underlying cellular processes responsible for the spontaneous events. Future research should focus on identifying patient-specific and ocular characteristics that predispose individuals to spontaneous MH closure, particularly in post-vitrectomy scenarios. Moreover, larger longitudinal studies are essential to explore the roles of Müller cells, cystoid macular edema, and the ERM in both the formation and closure of MHs. These studies should also assess the potential for non-surgical treatment options in select patients.
